# Transient dynamics and nonlinear fitness: A matrix approach to pulse and press perturbation

**DOI:** 10.1002/ecy.70433

**Published:** 2026-06-07

**Authors:** Harman Jaggi, Shripad Tuljapurkar, Wenyun Zuo, Samuel J. L. Gascoigne, Maja Kajin, Roberto Salguero‐Gómez

**Affiliations:** ^1^ Ecology and Evolutionary Biology Princeton University Princeton New Jersey USA; ^2^ Department of Biology Stanford University Stanford California USA; ^3^ School of Biological Sciences University of Aberdeen Aberdeen UK; ^4^ Department of Biology University of Oxford Oxford UK; ^5^ Department of Biology University of Ljubljana Ljubljana Slovenia

**Keywords:** matrix population models, nonlinear fitness, perturbation theory, press disturbance, pulse disturbance, the second derivatives of population growth rate, transient response matrix

## Abstract

Disturbances can occur as short‐lived pulses (e.g., storms) or sustained presses (e.g., chronic drought). Much work in ecology has developed methods to help predict how natural populations respond to disturbances, but analyses of pulse and press disturbances have been largely disconnected. We present a matrix framework that links press and pulse perturbations within the same analytical approach, showing how transient nonlinearities and demography shape fitness. We find that transient responses to pulse perturbations accumulate to determine the long‐term response to press disturbances. For structured population models, this cumulative change is given by a new transient response matrix (TRM). Strikingly, the TRM also yields the second derivatives of the population growth rate with respect to matrix elements. Thus, there is an intimate but unexpected relationship between nonlinear selection pressures on demographic rates, and the transient dynamics of populations. This relationship yields a strong correlation between TRM and generation time across 439 unique plant and animal species (2690 population models). We also show that the TRM is directly related to Cohen's cumulative distance measure for populations converging to stability. Our framework provides ecologists with a general tool to predict population responses to diverse environmental changes.

## INTRODUCTION

We live in a period of environmental disturbances that may push populations away from their historical states (Abbott et al., 2024; Barnosky et al., 2012). An important goal in ecology is to predict how natural populations respond to such changes (Morozov et al., 2024). Bender et al. (1984) introduced two terms (pulse and press) to distinguish between two types of experimental disturbances and to describe the combination of cause and effect (Glasby & Underwood, 1996). They classified disturbance regimes into acute, discrete events (pulse disturbances) and diffuse, sustained events (press disturbances).

A pulse disturbance is a one‐off event (such as an acute epidemic, or extreme but short weather event like a fire or hurricane) that may perturb the system away from its stable state if it fails to resist the disturbance. After a pulse, transient dynamics occur as the system returns to the previous stable state (Tao et al., 2021; White et al., 2013). Examples of a pulse disturbance include one‐off catastrophic events (Rypkema & Shripad, 2021), preferential targeting of prime adult individuals (Traill et al., 2014) in trophy hunting, droughts that primarily affect juveniles in plant populations (Refsland & Fraterrigo, 2018), among others. In contrast, a press disturbance is long‐lasting (e.g., global warming, land use change) and alters the stable state itself (Donohue et al., 2016; Inamine et al., 2022). In a press, there are also transient dynamics but the repeated disturbances push the population structure away from the original stable state distribution (hereafter, SSD) to the new stable state. Examples of a press perturbation include the decline in fertility rates of a population following chronic disease (Sironi, 2019), or the effects of reintroduction of a species into an ecosystem (Ripple & Beschta, 2012).

Much attention has been paid theoretically (Arnoldi et al., 2018; Hastings, 2001; Jentsch & White, 2019; Medeiros et al., 2025; Yang et al., 2008) and experimentally (Amor et al., 2020) to the responses of natural systems to pulse disturbances, and some to press disturbances (Inamine et al., 2022; Morozov et al., 2020). But previous work has not noted a direct connection between pulses and a press. In discrete‐time models, we consider a simplistic press perturbation to be a continuing series of pulse perturbations, one in every time interval. We use this equivalence to link transient response after a pulse to the response to a press.

To set the stage, we provide a brief overview of structured populations in discrete time, represented by a matrix population model (MPM) (Caswell, 2001). The MPM, denoted by B, has elements $b_{\mathrm{ij}}$ that describe the per capita contribution from state j to state i in one time step. These transition rates are nonnegative and may combine multiple vital rates (e.g., fertility is often the product of survival and fecundity). The long‐run growth rate of the population is given by the dominant eigenvalue of B and is denoted by $\lambda_{0}$. There are two eigenvectors associated with $\lambda_{0}$. The right eigenvector $u_{0}$ (i.e., ${\mathrm{Bu}}_{0}=\lambda_{0}u_{0}$) yields the stable stage distribution (SSD), that is, the proportion of individuals across states in the long run. The left eigenvector $v_{0}$ gives the stable reproductive value (SRV), which describes the contribution of individuals to future reproduction. Together, ($\lambda_{0},u_{0},v_{0}$) describe the asymptotic behavior of the population.

Now that we have described the long‐run behavior, we ask what happens when a small disturbance (such as a fire) shifts the system away from equilibrium. To examine this, we assume that the population is at its SSD and subject it to a one‐time pulse disturbance. We define a disturbance as changing the population's transition rates between states (ages or stages), and not as directly affecting population structure. Thus, at time t=0, the population is at $u_{0}$ and is impacted by a pulse between time t=0 and t=1. The result is that the subsequent population structure, at time t=1, is no longer the SSD, but is a sum, $u_{0}^{\prime }=\left(u_{0}+z^{*}\right)$, where the pulse produces the effect $z^{*}$ (see *Methods* section). The pulse does not recur (by definition), so its effect diminishes over time. So, the population structure at time t=2 is the sum of the SSD and the 1‐time‐diminished effect of the original pulse. Similarly, at time t=3, the population structure is the sum of the SSD and the 2‐time‐diminished effect of the original pulse. Eventually, the effect decays and the structure converges to the original stable structure $u_{0}$.

**FIGURE 1:**
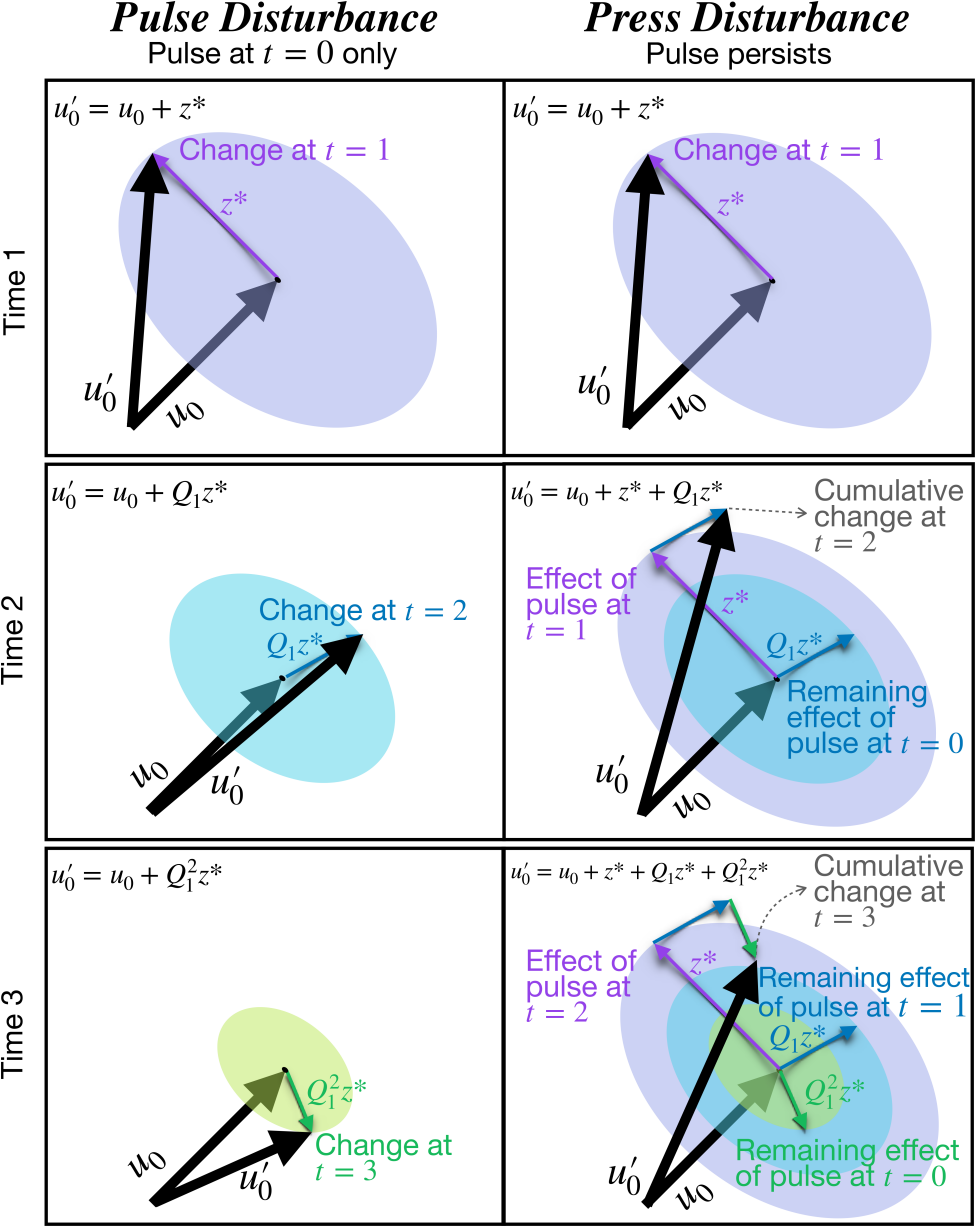
Dynamics of population structure after a pulse and press perturbation. The left panels illustrate the transient dynamics of a structured population following a single pulse disturbance at t=0. At time t=1, the purple arrow depicts the initial deviation $z^{*}$ from the stable distribution $u_{0}$. At time t=2, this single disturbance has decayed and rotated (blue arrow, $Q_{1}z^{*}$). At time t=3, it decays further (green arrow, $Q_{1}^{2}z^{*}$), eventually converging back to the original stable structure $u_{0}$. The right panels illustrate a press disturbance, which is modeled as a sequence of repeated pulses. The total deviation at any time step is the vector sum of the new pulse and the decayed remnants of all previous pulses. At t=1, the effect is identical to a single pulse (purple arrow, $z^{*}$). At t=2, the total displacement is the sum of the fresh pulse (purple arrow, $z^{*}$) plus the decayed effect of the pulse from t=1 (blue arrow, $Q_{1}z^{*}$). At t=3, the displacement is the sum of the fresh pulse (purple), the 1‐step decayed pulse (blue), and the 2‐step decayed pulse (green, $Q_{1}^{2}z^{*}$). The arrows are retained at each step to visualize how these historical effects accumulate to drive the population toward a new stable structure $u_{0}^{\prime }$.

**FIGURE 2:**
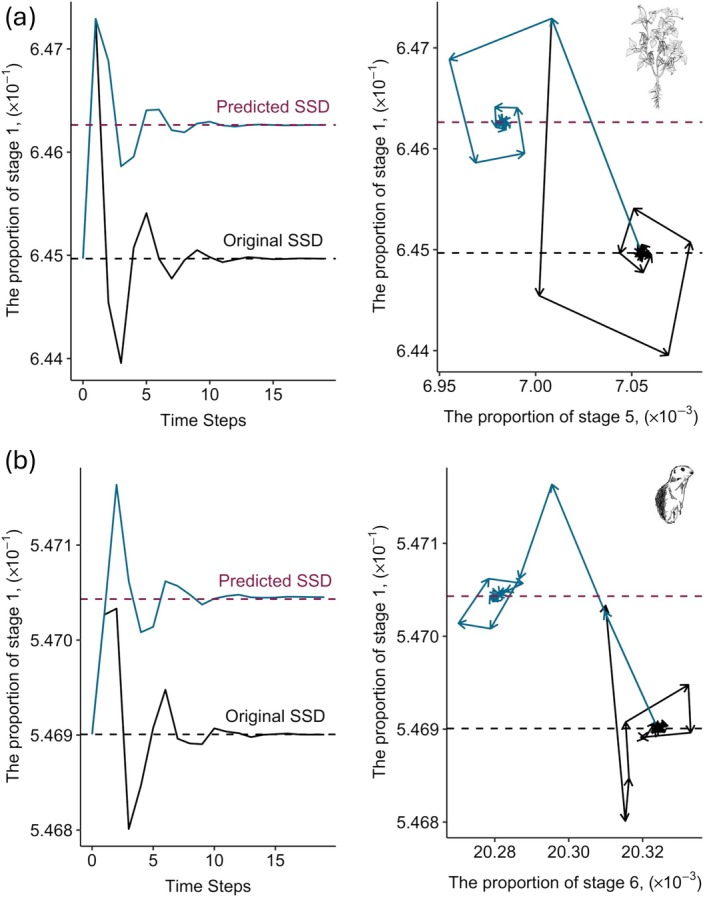
Comparing the response to pulse and press for *Phaseolus lunatus* and *Spermophilus dauricus*. The left panel of 2(a) shows transient dynamics for *Phaseolus lunatus* in Stage 1 after a pulse (black line) or a press (blue line) disturbance to the (1,6) and (6,5) elements of the matrix population model. The left panel of 2(b) shows transient dynamics for *Spermophilus dauricus* in Stage 1 after a pulse (black line) or a press (blue line) disturbance to the (1,7) and (7,6) elements of the matrix population model. In both panels, Stage 1 converges back to the original stable stage distribution (SSD; dashed black line) following a pulse disturbance, while a press disturbance leads to convergence toward a new SSD (dashed magenta line), analytically predicted using the Transient Response Matrix (TRM). On the right panel, the transient dynamics are visualized in a phase plane with Stage 5 on the *x*‐axis and Stage 1 on the *y*‐axis. Arrowheads indicate the direction of convergence. Both stages converge to the original SSD (black triangle) after a pulse disturbance, while a press disturbance drives convergence to a distinct SSD. Lima Bean sketch credit: IBPGR (1982) Daurian squirrel sketch credit: Harman Jaggi.

In contrast, the response to a press (sustained) perturbation (such as an ongoing drought) is an accumulation of responses to repeated pulses. The initial state is the SSD at time t=0, and a pulse between time 0 and 1 produces at time 1 the change $z^{*}$, exactly as before (as shown in Figure 1). At time t=2, however, we add together two effects: the 1‐time‐diminished effect of the first pulse (between time 0 and 1) and the next pulse (between time 1 and 2). At time t=3, we again add the new pulse to the remaining effect of previous pulses; the sum is the effect of the press disturbance. In the long‐term, we have a cumulative sum that yields the deviation from the original SSD to a new SSD; the cumulative sum is given by Equation (9), and illustrated in general by Figure 1. Numerical examples are in Figure 2.

Our framework shows how transient responses to pulse perturbations lead to a quantitative description of press perturbations. We derive the connection from pulses to presses via what we label a transient response matrix (TRM). We apply our framework to two stage‐structured MPMs (*Phaseolus lunatus* and *Spermophilus dauricus*) from the COMADRE and COMPADRE databases (Salguero‐Gómez et al., 2015, 2016). Using these examples, we show that small perturbations (pulse and press) leave distinct and predictable signatures on the population structure. These contrasting life histories also show how matrix structure shapes transient recovery and the range of perturbation over which our approximations are accurate. The response to a small pulse and press perturbation matches theoretical predictions (panel (a) and (b) in Figure 2). However, the resulting transient trajectories differ in shape and recovery time across the two life histories. Thus, our framework links experimental or observational perturbations to predicted consequences for long‐run growth and transient dynamics. For example, a researcher testing how invasive plants alter resource availability (press) or how an extreme frost event reduces survival in a single year (pulse) could use our approach to quantify both within the same modeling structure. The examples highlight the utility of our analysis in linking transient dynamics with long‐term demographic consequences.

Interestingly, the TRM also yields the second derivatives of the population growth rate with respect to demographic rates (matrix elements). Caswell (1996) and Shyu and Caswell (2014) have long argued for the importance of second derivatives and developed methods for their computation. While valuable, these methods have heavily relied on vector calculus—potentially obscuring important demographic processes connected to second‐order derivatives. Here we offer an intuitive method using perturbation theory, and our approach is valid for any discrete‐time, age‐, or stage‐based population model.

Previous work (Jiang et al., 2022) has identified a positive association between life‐history metrics and time to convergence. Based on previous macroecological work, we expect a correlation between TRM and generation time (average age of survival‐weighted reproduction). Using 439 unique species, we indeed find that a positive association between dominant (nonzero) eigenvalue of TRM and the generation time (in the *Results* section). In addition, the TRM is directly related to the cumulative distance to stability as defined by Cohen (1977). In the next section, we define MPMs and summarize (known) effects of pulse disturbances, and our approach to transient dynamics. Then we show precisely how pulses add to a press and how the responses to a press depend on the TRM, $J_{0}$.

## METHODS

Table 1 provides a summary of key definitions used for MPMs and its decomposition (as we discuss later in this section). As outlined in the Introduction, we consider structured population models in discrete time described by a MPM. The matrix may be a discretized version of an integral population model. The unperturbed MPM is denoted by B, and its elements $\left(b_{\mathrm{ij}}\right)$ are transition rates (per capita contributions of individuals) from state j at time t to state i at time $\left(t+1\right)$. The matrix B is nonnegative since transition rates ≥0. Starting with an initial population vector $n\left(0\right)$ and following the population at time t, $n\left(t\right)$, yields $n\left(t\right)=B^{t}n\left(0\right)$, over time t (Caswell, 2001).

**TABLE 1:** Symbols and definitions for matrix population models.

Symbol	Definition
B	Unperturbed matrix population model (MPM)
$b_{\mathrm{ij}}$	Transition rate from stage j to stage i
$\lambda_{0}$	Long‐run population growth rate: dominant eigenvalue of B
$u_{0}$	Stable stage distribution (SSD): right eigenvector of B
$v_{0}$	Stable reproductive value: left eigenvector of B
$n\left(t\right)$	Population vector at time t
$n\left(t\right)/\lambda_{0}^{t}$	Scaled population vector; converges to SSD over time.
$Q_{0}=u_{0}v_{0}^{T}$	Matrix that projects onto SSD
$Q_{1}=\left(B/\lambda_{0}-Q_{0}\right)$	Matrix that projects onto the non‐stable part of the structure
	So governs transients (matrix $Q_{1}$ is orthogonal to $Q_{0}$)

When the population projection matrix B is primitive and irreducible (as is typical for ecological models such as Leslie or Lefkovitch matrices), there is a dominant eigenvalue $\lambda_{0}$ with associated right and left eigenvectors, $u_{0}$ and $v_{0}$, respectively (Caswell, 2001). The dominant eigenvalue $\lambda_{0}$ determines the asymptotic population growth rate. The right eigenvector $u_{0}$ represents the stable stage distribution (SSD) toward which the population converges in the long term, and the left eigenvector $v_{0}$ defines the SRV describing their relative contribution to future population growth. The SSD $u_{0}$ is scaled so that its entries sum to one, expressing the proportion of individuals in each stage. This gives $e^{T}u_{0}=1$, where e is a vector of ones. The reproductive value vector $v_{0}$ is scaled so that the total reproductive value of the SSD equals one: $v_{0}^{T}u_{0}=1$. Here, T is transpose of a matrix.

We use two published MPMs from publicly available databases COMPADRE and COMADRE (Salguero‐Gómez et al., 2015, 2016). The first MPM is a 6‐stage annual plant for *Phaseolus lunatus* (Lima bean), with a population growth rate $\lambda_{0}=0.76$. The second is a 7‐stage mammalian matrix for *Spermophilus dauricus* (Daurian ground squirrel) with a population growth rate $\lambda_{0}=0.92$. In the *Results* section, we will use these contrasting life‐histories (an annual plant and a mammal) to illustrate how life‐history structure shapes transient recovery and demonstrate the generality of our results. The examples demonstrate how the same pulse or press perturbation can produce different transients and recovery times across life histories. The data and code are publicly available on link Zenodo.

### Decomposing population projection matrix into stable and transient components

To analyze transient and asymptotic behaviors, we rescale the original matrix by dividing by the population growth rate $\lambda_{0}$. Next, we decompose the rescaled matrix $\frac{B}{\lambda_{0}}$ into two parts:

(1) $$Q_{0}=u_{0}\;v_{0}^{T},$$

(2) $$Q_{1}=\left(\frac{B}{\lambda_{0}}-Q_{0}\right).$$

The matrix $Q_{0}$ comprises of stable vectors ($u_{0}$ and $v_{0}$) and projects any population vector onto the SSD, capturing the long‐term stable dynamics. Because $Q_{0}^{2}=Q_{0}$, it is a projection matrix. Moreover, the matrix $Q_{1}$ is orthogonal to $Q_{0}$ since $Q_{0}Q_{1}=Q_{1}Q_{0}=0$. This leads to the full decomposition:

(3) Bλ0=Q0+Q1.

Using this decomposition in Equation (3) and the fact that $Q_{0}$ and $Q_{1}$ are orthogonal, the population structure at time t, scaled by $\lambda_{0}^{t}$ is given by

$$\begin{array}{l} \frac{n\left(t\right)}{\lambda_{0}^{t}}=\frac{B^{t}n\left(0\right)}{\lambda_{0}^{t}}={\left(Q_{0}+Q_{1}\right)}^{t}n\left(0\right)\\ \;=Q_{0}n\left(0\right)+Q_{1}^{t}n\left(0\right) \end{array}$$

The term $Q_{0}n\left(0\right)=\left(v_{0},n\left(0\right)\right)\;u_{0}$ gives the asymptotic SSD since it is a scalar multiple of $u_{0}$. The term $Q_{1}^{t}n\left(0\right)$ governs the transient trajectory. This is because the spectral radius of $Q_{1}$ is less than one, and thus, the powers of $Q_{1}$ vanish over time, implying convergence to the SSD:

(4) $$\frac{n\left(t\right)}{\lambda_{0}^{t}}\to \left(v_{0},n\left(0\right)\right)\;u_{0}.$$

The decomposition in Equation (3) separates the stable ($Q_{0}$) and unstable ($Q_{1}$) components of population projection matrix B. This framework underpins recent ecological applications studying transient dynamics (Capdevila et al., 2021; Haridas & Tuljapurkar, 2007; Jiang et al., 2022; Koons et al., 2005).

### Transient dynamics after a pulse disturbance

Note that Table 2 summarizes the key definitions related to transient dynamics and perturbation analysis. Let us assume a population is initially at its SSD, $u_{0}$, with associated SRV vector $v_{0}$, and projection matrix B. If a pulse perturbation acts at t=0 for one time step, the population is no longer at equilibrium. To characterize the effects of this deviation, we use a decomposition based on the eigenstructure of B.

**TABLE 2:** Symbols and definitions for perturbation analysis.

Symbol	Definition
$z^{*}$	Perpendicular change to $u_{0}$ after a pulse disturbance
$Q_{1}^{t}z^{*}$	Transient dynamics after a pulse disturbance
D	Perturbation matrix, zero everywhere except perturbed element
$J_{0}=\frac{1}{\lambda_{0}}{\left(I-Q_{1}\right)}^{-1}\left(I-Q_{0}\right)$	Transient response matrix; captures cumulative effect of disturbance
$Z=J_{0}{\mathrm{Du}}_{0}$	Change in SSD due to a press disturbance
$Y^{T}=v_{0}^{T}{\mathrm{DJ}}_{0}$	Change in stable reproductive value due to a press
$s_{\mathrm{pq}}=\frac{\partial \lambda_{0}}{\partial b_{\mathrm{pq}}}$	Sensitivity of growth rate $\lambda_{0}$ to transition rate $b_{\mathrm{pq}}$
$\frac{\partial^{2}\lambda_{0}}{\partial b_{\mathrm{pq}}\partial b_{\mathrm{kl}}}$	Second derivative of growth rate with respect to $b_{\mathrm{pq}}$ and $b_{\mathrm{kl}}$
$\tau_{j}$	Inverse of damping ratio: $\tau_{j}=\frac{1}{e^{r_{0}-r_{j}}}$
$T_{c}$	Average age of survival‐weighted reproduction
$F\left(t\right)$	Cumulative distance to stability from initial structure

A small pulse perturbation modifies the matrix B to B+ϵD for one time step, where ϵ is the magnitude of pulse perturbation. The matrix D represents the matrix element being perturbed and is zero everywhere except at the entry ($D_{\mathrm{ij}}$) being perturbed. In ecological terms, this corresponds to a temporary change in a demographic rate. For example, perturbing the fertility rate $b_{15}$ would make the element $D_{15}=1$ as the only nonzero element of D. At time t=1, the population structure is no longer $u_{0}$, but instead becomes

(5) $$\hat{u}=u_{0}+z^{*},$$

where the shift $z^{*}$ lies in the transient subspace orthogonal to the SSD (derived in Appendix S1):

(6) $$z^{*}=\left(I-Q_{0}\right)\;\frac{D}{\lambda_{0}}u_{0}.$$

Because $z^{*}$ lies in the transient subspace, it determines how the population structure shifts from and eventually returns to the SSD. The proof for Equation (6) is given in Appendix S1: Section S1.2.

After the pulse ends, the population is again governed by the original matrix B and hence evolves according to

(7) $$\frac{B^{t}}{\lambda_{0}^{t}}\hat{u}=u_{0}+Q_{1}^{t}z^{*}.$$

As discussed previously:

1. The term $u_{0}$ remains constant—the population converges back to the $u_{0}$ because powers of $Q_{1}$ decay to zero.
2. The term $Q_{1}^{t}z^{*}$ evolves over time and captures the transient dynamics.

The path taken during the convergence to $u_{0}$ is determined by the transient component $z^{*}$ and its successive projections $Q_{1}^{t}z^{*}$. The full trajectory of the population structure through time (after a pulse) is the sequence:

$$u_{0}+z^{*},\;u_{0}+Q_{1}z^{*},\;u_{0}+Q_{1}^{2}z^{*},\ldots$$

Each step in this sequence represents the population structure at time t=1,2,3,… after the pulse. The rate and direction of convergence depend on the structure of $Q_{1}$ and the form of $z^{*}$. The term $Q_{1}^{t}z^{*}$ both shrinks in magnitude and may rotate in direction (as some directions may decay faster than others), depending on the eigenstructure of $Q_{1}$. Thus, $Q_{1}$ is the time‐dimished effect of the pulse produced by disturbance $z^{*}$ as discussed in the *Introduction*. We illustrate the key finding from this subsection in the left panel of Figure 1.

Our approach separates stable and transient components, offering a biologically interpretable approach to pulse dynamics. The research builds on and extends previous work on transient dynamics (Haridas & Tuljapurkar, 2007; Koons et al., 2017; Neubert et al., 2002; Stott, 2016).

### Press disturbance as a series of pulses

A press disturbance refers to a permanent change to the population projection matrix B, represented as B+ϵD, where ϵ is the magnitude of perturbation and D is a matrix with nonzero entries for elements that perturbed. We assume the perturbation is small, so that first‐ and second‐order approximations apply. A key idea is that a press disturbance is equivalent to an ongoing sequence of identical pulse perturbations.

We begin by characterizing the response to a single pulse, which shifts the structure by a vector $z^{*}$ perpendicular to $u_{0}$, as described in Equation (6) given by $z^{*}=\epsilon \left(I-Q_{0}\right)\frac{D}{\lambda_{0}}u_{0}$. This vector satisfies $Q_{0}z^{*}=0$ and captures the deviation from the SSD caused by the disturbance.

Because a press perturbation acts at each time step, the full trajectory of the population structure consists of accumulating transient effects and new perturbations. At time t=1, the structure is $u_{0}+z^{*}$ (same as pulse disturbance). Now at t=2, we apply the new perturbation and add the decayed contribution of the previous perturbation:

(8) $$\frac{n\left(2\right)}{\mu^{2}}=u_{0}+z^{*}+Q_{1}z^{*},$$

where μ is the new dominant eigenvalue under the press disturbance. That is, μ is the dominant eigenvalue of the B+ϵD. Continuing in this way, we apply the new perturbation and add the decayed contribution of the previous perturbations to get the general expression:

(9) $$\frac{n\left(t\right)}{\mu^{t}}=u_{0}+z^{*}+Q_{1}z^{*}+Q_{1}^{2}z^{*}+\ldots +Q_{1}^{t-1}z^{*}.$$

We illustrate this approach in the right panel of Figure 1. As time increases (t→∞), the cumulative effect converges to a geometric series:

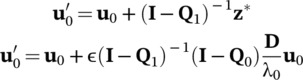

where $u_{0}^{\prime }$ represents the new stable stage distribution (SSD) under the press disturbance. Similarly (as discussed in Appendix S1: Section S2), an analogous argument can be made for the SRV.

Next, we define the TRM as follows:

(10) J0=ϵ1λ0I−Q1−1I−Q0.

Then the change in SSD (up to second order) from the press disturbance becomes:

(11) u0′≈u0+J0Du0.

These results provide an approximation for the effects of press disturbances on the long‐term population structure and reproductive values. The approximation for $u_{0}^{\prime }$ and $v_{0}^{\prime }$ is exact in the limit of small perturbations since higher order terms can be neglected. This means that we have obtained the linear change (equivalently, the first derivative) of the SSD (or SRV).

Our approach uses only the dominant eigenstructure and the TRM $J_{0}$ thereby generalizing the analysis in Caswell (1996) and making it broadly applicable to structured population models. The transient response matrix $J_{0}$ maps small changes in the projection matrix to changes in the SSD (or SRV). The TRM $J_{0}$ describes the accumulation of the effects of continued disturbance, as well as the intrinsic ability to dampen the perturbations. In a later section, we discuss the many applications of the new matrix $J_{0}$.

We illustrate the dynamics of pulse (left panel) and press disturbances (right panel) in Figure 1, which shows how transient deviations decay and evolve over time using colored time‐diminished vectors. Further, we test our theory on two life histories as shown in two panels in Figure 2 in the *Results* section.

### Quantifying change in fitness: Sensitivity and nonlinear response

In MPMs, fitness is the long‐run growth rate $\lambda_{0}$ determined by the elements of matrix B. Ecologists have long studied the sensitivities $s_{\mathrm{pq}}$ of fitness (Caswell, 2001) with respect to each matrix element $b_{\mathrm{pq}}$. However, fitness may also vary nonlinearly with respect to one or more matrix elements, motivating our analysis of second‐order derivatives. The second derivatives of population growth rate measure the curvature of fitness with respect to changes in vital rates (Caswell, 1996). They quantify whether the combined effect of simultaneously changing two rates is approximately additive (weak curvature) or shows nonadditive effects (strong curvature). This matters when perturbations affect multiple stages at once, or when vital rates covary due to environment or life‐history trade‐offs. In the next section, we begin with a simple intuitive approach to sensitivity (first‐order fitness response) and then use it to study the second derivative (or curvature) of population growth to matrix perturbations.

#### Linear change and sensitivity

Consider a stable population at the SSD, with a fraction $u_{0q}$ of individuals in stage q, and a one‐period population growth rate of $\lambda_{0}$. Hence, $u_{0q}$ is the fraction of individuals available to make a q→p transition. Since $b_{\mathrm{pq}}$ is the rate for that transition, the number of individuals in state p produced by that transition is proportional to the product $u_{0q}\;b_{\mathrm{pq}}$. In that final state, every individual has reproductive value $v_{0p}$, so the product

$$v_{0p}\;b_{\mathrm{pq}}\;u_{0q}$$

is the relative contribution of the q→p transition to population growth. The total of these contributions over all initial and final states is $\lambda_{0}$ (as it should be).

Now suppose that we add a small amount $\partial b_{\mathrm{pq}}$ to the rate for the q→p transition, that is, we change $\left(b_{\mathrm{pq}}\right)$ to $\left(b_{\mathrm{pq}}+\partial b_{\mathrm{pq}}\right)$. Following the logic above, the change in growth rate is the product of three terms:

1. The fraction of population that is subject to this state change, that is, $u_{0q}$.
2. The change in that transition rate, that is, $\partial b_{\mathrm{pq}}$.
3. The relative “value” of an additional contribution to final state q, that is, $v_{0p}$.

The product is $\partial b_{\mathrm{pq}}\;v_{0p}\;u_{0q}.$ Dividing by $\partial b_{\mathrm{pq}}$, we conclude that the first derivative of population growth rate is

(12) $$\frac{\partial \lambda_{0}}{\partial b_{\mathrm{pq}}}=v_{0p}\;u_{0q}=s_{\mathrm{pq}}.$$

Here, $s_{\mathrm{pq}}$ is the sensitivity with respect to $b_{\mathrm{pq}}$ element and the last equality is the standard result (Caswell, 2001). We now use our new simple method to find the second derivatives of fitness.

#### Second‐order fitness response to matrix perturbation

**FIGURE 3:**
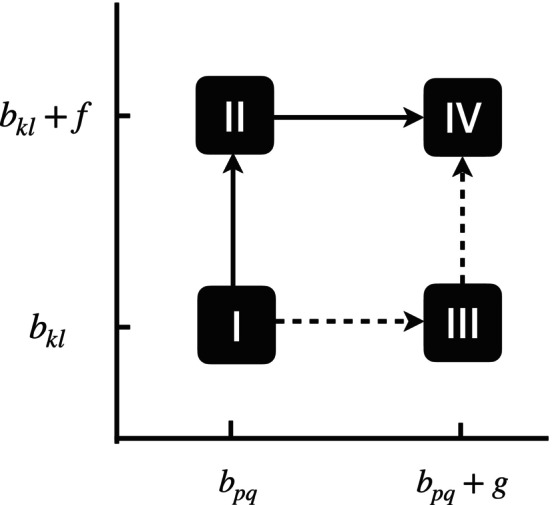
Computing the second derivatives of fitness with respect to matrix elements. The horizontal and vertical axes indicate rates for the two demographic transitions, $\left(p,q\right)$ and $\left(k,l\right)$. Point I indicates the starting values, where the fitness is $\lambda_{0}$, SSD is $u_{0}$ and stable reproductive value is $v_{0}$. A press disturbance of both demographic rates ends at point IV. We consider two possible routes. First route: Go from I to II by changing only the demographic rate for the k←l transition (i.e., $b_{\mathrm{pq}}$ is unchanged but $b_{\mathrm{kl}}$ becomes $b_{\mathrm{kl}}+f$). At II the new stable population is, say, $u_{0}+Z_{1}$ and the new reproductive value is, say, $v_{0}+Y_{1}$. Next go from II to IV, by changing only $b_{\mathrm{pq}}$ by an amount g. Second route: Starting at I, go from I to III by perturbing the p←q transition rate by an amount g. At III the new stable population is, say, $u_{0}+Z_{2}$ and the new reproductive value is, say, $v_{0}+Y_{2}$. Next, go from III to IV by changing only $b_{\mathrm{kl}}$ by an amount *f*.

Suppose a small press disturbance affects the $b_{\mathrm{pq}}$ and $b_{\mathrm{kl}}$ elements of the matrix B, where p,q,k,l are indices. For example, a perturbation to the fertility element $b_{15}$ would represent a shift in offspring production, whereas a perturbation in survival element $b_{21}$ would correspond to a change in state survival. Figure 3 illustrates two sequences of applying these changes. In the first path, we modify the $\left(\mathrm{kl}\right)$ transition rate by f (I → II), followed by the $\left(\mathrm{pq}\right)$ rate by g (II → IV). In the second path, we reverse the order: modify $\left(\mathrm{pq}\right)$ first (I → III), then $\left(\mathrm{kl}\right)$ (III → IV). Both routes must yield the same total change in fitness, as fitness is a scalar function of matrix entries.

We first consider the I → II → IV path. At point I, the SSD is $u_{0}$ and the SRV is $v_{0}$. Using the logic from the previous subsection, the fitness change in the I → II step is

$$f\;v_{0k}\;u_{0l}.$$

At point II, the SSD has changed from $u_{0}$ to $u_{0}+Z_{1}$, where $Z_{1}$ quantifies the first‐order change in SSD due to the perturbation in $b_{\mathrm{kl}}$ (as discussed in Appendix S1: Section S2):

(13) $$Z_{1}=f\left(\begin{array}{c} J_{0,1k}u_{0l}\\ J_{0,2k}u_{0l}\\ \vdots \end{array}\right).$$

Also at II, the SRV is $\left(v_{0}+Y_{1}\right)$ with

(14) $$Y_{1}^{T}=g\left(J_{0,l1}v_{0k}\;J_{0,l2}v_{0k}\;\ldots \right).$$

So in the transition II to IV, the fitness changes by the product of

1. The stable proportion in stage q, which Equation (13) shows is $\left(u_{0q}+Z_{1q}\right)$.
2. The change in the rate, g.
3. The SRV in stage p, which Equation (14) shows is $\left(v_{0p}+Y_{1p}\right)$.

Thus the total change in the presses from I to II, and II to IV (see Figure 3) is

$$\begin{array}{c} f\;v_{0k}\;u_{0l}+g\left(v_{0p}+Y_{1p}\right)\left(u_{0q}+Z_{1q}\right)\\ \approx f\;v_{0k}\;u_{0l}+g\;v_{0p}\;u_{0q}+g\;\left(u_{0q}\;Y_{1p}+v_{0p}Z_{1q}\right), \end{array}$$

where the higher‐order term $gY_{1p}Z_{1q}$ is ignored because it is close to zero. Split this up into two bits

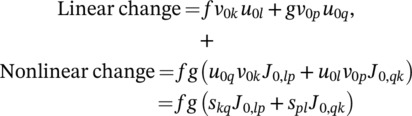




When f and g are small, the nonlinear change yields the second derivatives of fitness:

(15) ∂2λ0∂bpq∂bkl=splJ0,qk+skqJ0,lp

where we use the sensitivities $s_{\mathrm{kq}}=v_{0k}\;u_{0q}$ and $s_{\mathrm{pl}}=v_{0p}\;u_{0l}$. This expression for the second derivative is symmetric with respect to an exchange of the elements $b_{\mathrm{pq}},b_{\mathrm{kl}}$ (as it should be). The curvature of fitness is measured by the second derivatives in Equation (15) and depends on both the first‐order sensitivities and the TRM ($J_{0}$). As shown in Figure 3, we could alternatively go from I to III and then III to IV. That process involves different changes to the SSD and reproductive value but yields the same final result. The details of this calculation are presented in the Appendix S1: Section S2.3. In Appendix S1: Section S2.4, we provide a stepwise calculation of second derivatives for *Phaseolus lunatus*. Our calculation for the final answer exactly matches Shyu and Caswell (2014); however, our approach is more intuitive and based on application of perturbation theory. A complete derivation can be found in Appendix S1: Section S2.2.

### Transient response matrix $J_{0}$

The previous two sections identify the central role of the TRM $J_{0}$ in the linear and nonlinear responses to a disturbance, where

$$J_{0}=\frac{1}{\lambda_{0}}{\left(I-Q_{1}\right)}^{-1}\;\left(I-Q_{0}\right).$$

Since matrix $Q_{1}$ shapes the transient dynamics after a pulse, those transient dynamics also shape the TRM, and thus the nonlinear change in growth rate. This connection between transients and the nonlinearity of the dominant eigenvalue is unexpected and intimate. Here, we further illuminate this connection by three new results that connect the TRM to transients.

#### TRM and cumulative distance to stability

In general, even when the MPM does not have distinct eigenvalues, an important metric to assess the difference between an observed stage distribution and the SSD is the cumulative distance to stability (Cohen, 1979a). Cumulative distance to stability measures the total displacement experienced while a population returns to its stable stage structure after a perturbation (Cohen, 1977). It captures both the size and duration of transient shifts in stage composition. Ecologically, this is important because two populations can have the same long‐run growth rate but different cumulative distances and therefore different short‐term risks and management consequences. Several studies (White et al., 2013; Williams et al., 2011) have employed this metric and here we show that the TRM is closely related to the cumulative distance.

Any non‐stable initial population distribution at time t=0 converges toward the SSD. Following Cohen (1979a), at each time t>0, the “distance” from stability is measured by summing the elements of the difference vector $f\left(t\right)=\lambda_{0}^{-t}\;n\left(t\right)-Q_{0}\;n\left(0\right).$ Convergence means that this distance is decreasing, so the cumulative vector $F\left(t\right)=\sum_{m=0}^{\left(t-1\right)}\;f\left(m\right)$ should have a limit. For an initial population vector $n\left(0\right)$ Cohen showed this limit to be

(16) $$\lim_{t\to \infty }\;F\left(t\right)=\left[{\left(I+Q_{0}-\frac{B}{\lambda_{0}}\right)}^{-1}-Q_{0}\right]n\left(0\right).$$

But we find (see Appendix S1: Section S4 for matrix equivalence) that Cohen's cumulative distance is just

(17) $$\lim_{t\to \infty }\;F\left(t\right)=J_{0}\;n\left(0\right).$$

Thus, the TRM $J_{0}$ is directly related to the asymptotic cumulative distance to stability.

#### TRM for population matrices with distinct eigenvalues

Suppose that the MPM B has distinct eigenvalues, so that $u_{j},v_{j}$ are the right, left eigenvectors for eigenvalue $\lambda_{j}=e^{\left(r_{j}+i\omega_{j}\right)}$ for j≥1. Below, superscript † indicates a complex conjugate transpose.

Define the eigenvalue ratios

$$\frac{\lambda_{j}}{\lambda_{0}}=\frac{e^{\left(r_{j}+i\omega_{j}\right)}}{e^{r_{0}}}=\tau_{j}\;e^{i\omega_{j}}$$

where $\tau_{j}=e^{r_{j}-r_{0}}$ and $\tau_{j}<1$; the $\tau_{j}$ are the inverse damping ratios Caswell (2001).

Then a spectral decomposition and the definitions (1) and (2) yield the expression:

(18) $$Q_{1}=\sum_{j\geq 1}\;\tau_{j}\;e^{i\omega_{j}}\;u_{j}\;v_{j}^{†}.$$

From this, we can now expand $J_{0}$ (see Appendix S1: Section S3) as

(19) $$J_{0}=\sum_{j\geq 1}\;\frac{u_{j}\;v_{j}^{†}}{\left(1-\tau_{j}\;e^{i\omega_{j}}\right)}.$$

This is a spectral decomposition of the TRM (excluding the eigenvector $u_{0}$ for which the eigenvalue is 0). Thus, the vectors $u_{j}$, $v_{j}$ are right, left eigenvectors of $J_{0}$ corresponding to eigenvalue ${\left(1-\tau_{j}\;e^{i\omega_{j}}\right)}^{-1}$. One consequence is that the dominant nonzero eigenvalue of the TRM depends on the damping ratio $\tau_{1}$, being low when $\tau_{1}\ll 1$ and becoming large when $\tau_{1}$ approaches 1.

## RESULTS

To test our framework and evaluate how structured populations respond to disturbances, we analyze both short‐term (pulse) and long‐term (press) perturbations for two population projection matrices. Our first result shows that pulses and presses leave distinct signatures on population structures and can be predicted using our method. Next, we examine how the TRM ($J_{0}$) relates to life‐history traits like generation time ($T_{c}$), defined as average age of survival‐weighted reproduction. Finally, we demonstrate how first‐ and second‐order derivatives of population growth rate vary in direction and magnitude. Together, our results show how transient dynamics are linked to long‐term outcomes and life‐history traits via transient repsonse matrix. Note that a table of key vectors and matrices with their definitions is provided in Tables 1 and 2.

### Convergence to stable stage under press and pulse disturbances for two life‐histories: *Phaseolus lunatus* and *Spermophilus dauricus*

To examine how press and pulse disturbances shape population structure over time, we simulate perturbations for two life histories: *Phaseolus lunatus* (Lima bean), an annual plant with a 6‐stage MPM, and *Spermophilus dauricus* (Daurian ground squirrel), a mammal represented by a 7‐stage MPM. The MPMs are taken from open source COMPADRE and COMADRE databases, respectively (Salguero‐Gómez et al., 2015, 2016). We focus on disturbances applied to fecundity and survival rates, specifically elements (1,6) and (6,5) for Lima bean; (1,7) and (7,6) for Daurian ground squirrel (panel (a) and (b) in Figure 2). The perturbations reflect environmental shocks that have one‐time (pulse) or persistent effects (press) on the two life histories.

In the left panel of Figure 2, we track the proportion of individuals in Stage 1 over time following either a single‐time pulse (black line) or a sustained press (blue line) disturbance (increased each by 2%). The system returns to the unperturbed stable stage distribution (SSD) after a pulse, as expected from matrix analysis discussed above. In contrast, the press disturbance leads to a new equilibrium structure, indicated by the magenta dashed line. The new SSD is accurately predicted by the TRM $J_{0}={\left(I-Q_{1}\right)}^{-1}\left(I-Q_{0}\right)$ using perturbation theory, reinforcing its utility in forecasting stable st(ages) under sustained environmental pressures.

The right panel projects the same dynamics onto a phase plane composed of Stage 1 and Stage 5 proportions for Lima bean; Stage 1 and Stage 6 for Daurian ground squirrel. Both trajectories exhibit damped convergence—either toward the original SSD (black triangle) for the pulse or a new SSD under press, confirming theoretical expectation on how the long‐run structure shifts under press perturbations. The convergence trajectories spiral back to stable state, revealing differences in the strength and shape of transient dynamics across the two life‐histories.

We show numerically that even moderate perturbations to MPMs yield excellent agreement between predicted and observed $u_{0}^{\prime }$. Our result illustrates how small, short‐lived disturbances can generate complex trajectories in stage structure before long‐term convergence as shown in Figure 2.

### Linking TRM to life‐history traits

**FIGURE 4:**
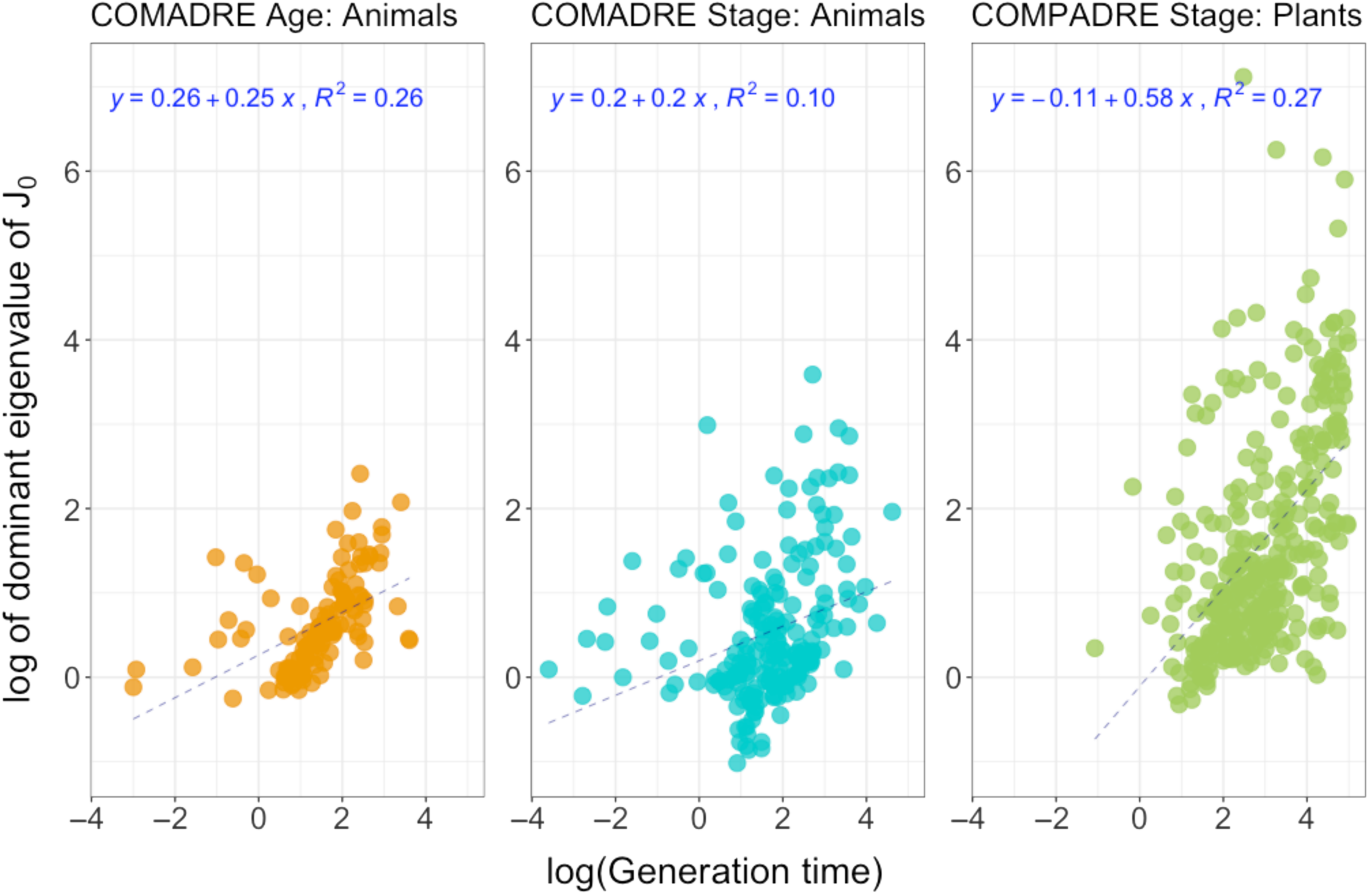
Relationship between generation time and transient response matrix. The figure examines the relationship between generation time ($T_{c}$) and the dominant eigenvalue of Transient Response Matrix ($J_{0}$) based on phylogenetic generalized least squares. The *y*‐axis corresponds to log of the dominant eigenvalue of $J_{0}$ and the *x*‐axis corresponds to log of generation time $T_{c}$. Each panel corresponds to a database: COMADRE age‐structured matrices, COMADRE stage‐structured matrices, COMPADRE stage‐structured matrices. The correlation between dominant eigenvalue of $J_{0}$ and $T_{c}$ is consistently positive across all databases.

Jiang et al. (2022) found a strong correlation between damping time τ and generation time ($T_{c}$), a key life‐history trait (Gaillard et al., 2005). Given the association between damping time and generation time, we expected the dominant nonzero eigenvalue of TRM ($J_{0}$) to scale $T_{c}$. To examine this scaling, we analyzed 439 unique age and stage‐ structured species (after correcting for phylogenetic inertia) using the COMADRE Animal Matrix Database (Salguero‐Gómez et al., 2016), the COMPADRE Plant Matrix Database (Salguero‐Gómez et al., 2015), and previously published mammalian database in Jiang et al. (2022). As shown in Figure 4, we find that the dominant nonzero eigenvalue of $J_{0}$ is strongly correlated with generation time $T_{c}$ on the log–log scale.

This relationship can be understood as follows: Species with longer generation times exhibit slower convergence to their stable distribution following a perturbation. Since the TRM $J_{0}$ captures the cumulative effect of transient dynamics under disturbance, its dominant eigenvalue effectively summarizes the timescale of decay toward equilibrium. A large generation time $T_{c}$ implies that demographic transitions are inherently slower, which delays convergence and amplifies the transient contribution.

### Comparing range of sensitivities, TRM and second‐order derivatives of population growth rate

**FIGURE 5:**
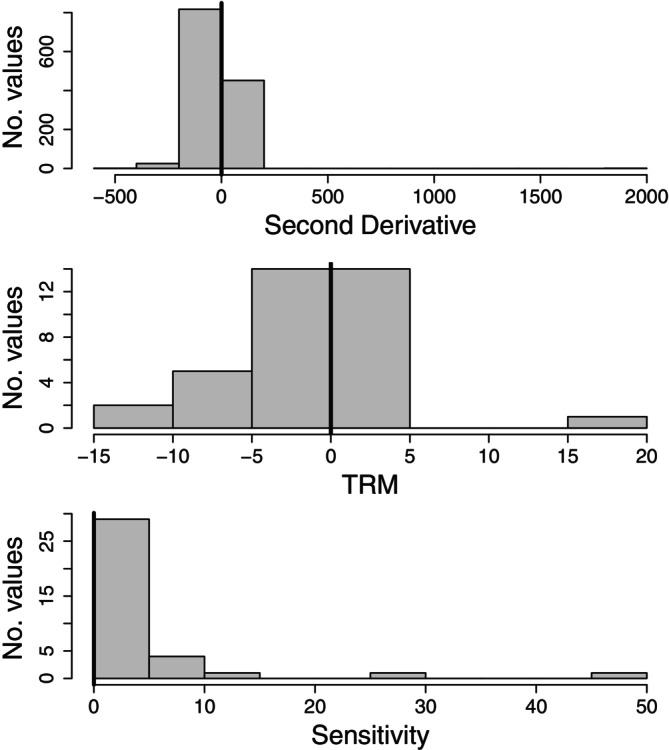
Histograms to show range and distribution in values of second derivatives, transient response matrix ($J_{0}$), and sensitivities. Second derivative of population growth rate and $J_{0}$ both have positive and negative values, but sensitivity of growth rate only have positive values.

We examine the range and distribution of key quantities: the second derivatives of population growth rate, the TRM ($J_{0}$), and first‐order sensitivities for *Phaseolus lunatus*. As shown in Figure 5, both $J_{0}$ and the second derivatives exhibit a wide spread of values, spanning positive and negative ranges. Ecologically, this means the fitness response to perturbations can be locally concave or convex: second‐order terms can either dampen or amplify the first‐order effect of a change in a vital rate. Thus, curvature captured by the second derivative can be positive or negative depending on the demographic pathway of the perturbation, that is, which demographic rates are perturbed and how they covary.

In contrast, the first‐order sensitivities are strictly nonnegative, because all terms in $s_{\mathrm{pq}}=v_{0p}u_{0q}$ are always positive for population projection matrices. The new insight from second derivatives and TRM is that they capture the nonlinear and potentially deteriorating effect of perturbations. This is because the curvature, captured by the second derivative of population growth rate can be positive or negative based on effect of perturbation. Thus, second derivatives and TRM extend classical sensitivity analysis, emphasizing the importance of second‐order terms in forecasting demographic responses to disturbance.

## DISCUSSION

Population ecology has made important progress in understanding how natural populations respond to one‐off (i.e., pulse) disturbances (Jentsch & White, 2019; Tao et al., 2021). Here, we developed a novel approach to examine how natural populations respond to press disturbances, which can be expressed as repeated pulses, when the disturbances are small. Our results reveal an intimate and unexpected connection between the nonlinearity of population growth rate to vital rates, and transient dynamics. We apply our theoretical predictions to *Phaseolus lunatus* and *Spermophilus dauricus* and find that both pulse and press disturbances have distinct and predictable consequences on the population structure. These examples reinforce the value of our framework across life‐histories and complements recent work on demographic resilience (Dakos & Kéfi, 2022; Kunze et al., 2025; Levine et al., 2024; MacDonald et al., 2024), which emphasizes the role of transient dynamics in buffering populations. It is important to note that this work does not consider general time‐dependence or full‐continuum of intensity‐frequency trade‐offs as examined in disturbance theory in ecology (Burton et al., 2020; Gough et al., 2024; Miller et al., 2011). Instead, our focus is to analyze a specific and analytically tractable limiting case where a persistent or press perturbation can be represented as an accumulation of repeated pulses.

Our analysis leads to a new matrix TRM ($J_{0}$) which predicts structure shifts following a press disturbance. When a pulse disturbance acts on a structured population, the resulting transient dynamics are characterized by the damping matrix $Q_{1}$. From a macroecological perspective, we find further evidence on how demography shapes resilience (Capdevila et al., 2020; Jiang et al., 2022). First, the largest eigenvalue of TRM is positively correlated with generation time ($T_{c}$). Second, the TRM is in fact essentially equal to the cumulative distance to stability after a pulse, as defined by Cohen (1979a). The work provides an explicit algebraic link between pulses, and sustained press perturbations. We focus on small disturbances, similar to what is done for a linear sensitivity analyses. However, in MPMs, a small press produces a linear change in the SSD but a nonlinear change in growth rate (fitness), and thus our approach may be akin to a nonlinear sensitivity analysis. Our approach should help researchers examine impacts of linear and nonlinear selection on vital rates such as survival, growth, and reproduction.

The second derivatives of population growth rate have evolutionary implications (Brodie et al., 1995; Doak et al., 1994; Shyu & Caswell, 2014; Vasseur & Fox, 2007). In evolutionary ecology, selection acts on vital rates through their effects on fitness, and curvature in $\lambda_{0}$ determines how variation in vital rates translate into changes in long‐run growth. The second derivatives quantify nonadditive effects, which can shape the strength and direction of selection when multiple traits (in our case matrix elements) covary. Phillips and Arnold (1989) refer to negative curvature (concave) as stabilizing selection on a rate, positive curvature (convex) as disruptive selection, and mixed second derivatives as correlational selection as selection may favor increases in two rates together versus a trade‐off where one increases as the other decreases. In particular, they reveal (1) whether the average fitness of individuals in the population changes linearly as vital rates are perturbed (e.g., Caswell 1996); (2) if fitness is nonlinear, whether fitness is concave or convex (Kajin et al., 2025); and (3) if the second derivative of fitness with respect to vital rates is positive for observed life histories, then the observed value corresponds to a local minimum, and vice versa (Brodie et al., 1995). Here, property (4) may be particularly useful when examining selection for optimal life‐history strategies (Charlesworth, 1994).

We consider structured populations described by a MPM in a constant environment, but the analysis may be generalized to other contexts, such as community composition and nonlinear multispecies interactions (Bender et al., 1984; Collins et al., 2020; Ratajczak et al., 2017). For example, suppose we have a population vector n following a nonlinear MPM with matrix $A\left(\theta ,n\right)$ with parameters θ, and dynamics

$$n\left(t+1\right)=A\left(\theta ,n\right)\;n\left(t\right),$$

and there is an equilibrium population $n^{*}$ that is locally stable. Here of course, vector $n\left(t\right)$ may have components that are stages of one species or of many species. Now consider a press that changes the parameters to θ to $\left(\theta +\epsilon \phi \right)$. Then the original stable equilibrium changes to say $n^{*}+x$, and our methods can be used directly to compute the change x. We plan to follow this direction of analysis in later work.

Our findings are also relevant for stochastic population dynamics and linear response theory (De Nittis & Lein, 2017; Jaggi, Steinsaltz, & Tuljapurkar, 2024; Ruelle, 2009). However, these results in a stochastic context also require that the noise be small. In particular, a population in a stochastic environment can be viewed in terms of a sequence of unequal pulses and their cumulative effects. So, for example, we can examine how shifts in mean, variance, and temporal autocorrelation may impact a population's ability to persist (Drake, 2005; Vasseur & Fox, 2007). Indeed, we note that the long‐run stochastic growth rate in a serially correlated environment is given by a quantity that is quite similar to the TRM of this paper, but extended to include the stochastic autocorrelation in the environment (Tuljapurkar & Haridas, 2006). We also expect that our methods may be useful in studying the interplay between pulse and press disturbances while incorporating stochastic growth rates (Caswell, 2010; Jaggi, Steinsaltz, & Tuljapurkar, 2024), density dependence (Jaggi, Zuo, et al., 2024; Travis et al., 2023), or nonlinear interactions within ecological communities (Medeiros et al., 2023; Medeiros & Saavedra, 2023).

Our approach complements earlier work on MPMs. Some general results are found in Cohen's work on the convexity of the dominant eigenvalue (Cohen, 1979a, 1979b, 1980, 1981) used by, for example, Drake (2005), with more recent work by McCarthy et al. (2008) and Stott (2016). We hope these insights into the connections between nonlinearity, transients, and selection pressures will be useful in future research on understanding and managing population dynamics in a changing world.

## CONFLICT OF INTEREST STATEMENT

The authors declare no conflicts of interest.

## Supporting information


Appendix S1.

## Data Availability

Data and code (Jaggi et al., 2026) are available in Zenodo at https://doi.org/10.5281/zenodo.18901258.
