# Supplementary material for: Transient dynamics and nonlinear fitness: A matrix approach to pulse and press perturbation
Source: Ecology. 2026 Jun 7;107(6):e70433. doi: 10.1002/ecy.70433 (PMC13243821; doi:10.1002/ecy.70433)
Supplement: Supplementary file 1 — Appendix S1. [file ECY-107-e70433-s001.pdf]

Appendix S1

Transient dynamics and nonlinear fitness: A matrix approach to pulse and press perturbation

Harman Jaggi, Shripad Tuljapurkar, Wenyun Zuo, Samuel J. L. Gascoigne,

Maja Kajin, and Roberto Salguero-Gómez

Ecology

Appendix S1 3

Section S1 Pulse Disturbances and Transients 3

S1.1 Basic Decompositions 3

S1.2 The perpendicular part of population structure following pulse . . 3

Section S2 Perturbation theory for a Press disturbance 5

S2.1 Second Order Perturbation: finding η_2 6

S2.2 Second order derivatives 8

S2.3 The alternative route to calculate the second derivatives 10

S2.4 Stepwise Calculation of Second derivatives for *Phaseolus lunatus* 12

Section S3 When the population matrix has distinct eigenvalues 16

Section S4 Relation between TRM and Cohen's cumulative distance 18

Section S1: Pulse Disturbances and Transients

S1.1 Basic Decompositions

We decompose the population matrix as in the main text. Note that

$$\mathbf{Q}_0\mathbf{u}_0 = \mathbf{u}_0,\ \mathbf{Q}_0^2 = \mathbf{Q}_0.$$

$$\text{and } \mathbf{Q}_1 = \frac{1}{\lambda_0}\left(\mathbf{B} - \lambda_0\,\mathbf{Q}_0\right) \tag{S1}$$

S1.2 The perpendicular part of population structure following pulse

Suppose a pulse disturbance modifies the matrix **B** to **B** + **D** for one time step at $t = 0$. The matrix **D** is assumed to be zero everywhere except at the entry being perturbed. Since this is a pulse disturbance, the projection matrix reverts to **B** for the next time steps. Here, we assume that the perturbation is small enough so that normalization to total size is not needed. After the pulse, the population structure becomes:

$$\widehat{\mathbf{u}} = \frac{(\mathbf{B} + \mathbf{D})\mathbf{u}_0}{\lambda_0} = \mathbf{u}_0 + \frac{\mathbf{D}}{\lambda_0}\mathbf{u}_0 = \mathbf{u}_0 + \mathbf{z}.$$

where $\mathbf{z} = \frac{\mathbf{D}\mathbf{u}_0}{\lambda_0}$ is the shift in population structure caused by the pulse disturbance. However, we are interested in the transient part of the new structure, that is the part that eventually dies out. Such a vector would be orthogonal to SSD. Any part of $\mathbf{z}$ aligned with $\mathbf{u}_0$ is only a rescaling of all stages and thus does not represent a change in population structure.

Let $\mathbf{z}^*$ be the component of $\mathbf{z}$ that lies entirely in the transient subspace (i.e., orthogonal to $\mathbf{u}_0$ in the sense of the left eigenvector $\mathbf{v}_0$). To extract the transient component, we want a vector $\mathbf{z}^*$ that is not aligned with stable component $\mathbf{Q}_0$. We achieve this by subtracting from $\mathbf{z}$ its component along $\mathbf{u}_0$. That is-

$$\mathbf{z}^* = \mathbf{z} - \mathbf{Q}_0\mathbf{z} = (\mathbf{I} - \mathbf{Q}_0)\mathbf{z}.$$

Applying this projection $(\mathbf{I} - \mathbf{Q}_0)$ to $\mathbf{z}$ yields the orthogonal or transient component:

$$\boxed{\mathbf{z}^* = (\mathbf{I} - \mathbf{Q}_0)\,\frac{\mathbf{D}}{\lambda_0}\,\mathbf{u}_0,}$$

which is Equation (6) in the main manuscript. Note that the matrix $(\mathbf{I} - \mathbf{Q}_0)$ satisfies:

$$(\mathbf{I} - \mathbf{Q}_0)\mathbf{u}_0 = 0, \quad \text{and} \quad \mathbf{v}_0^T(\mathbf{I} - \mathbf{Q}_0) = 0.$$

This orthogonality is given by $\mathbf{v}_0^T\mathbf{z}^* = 0$, or equivalently $\mathbf{Q}_0\mathbf{z}^* = 0$.

Section S2: Perturbation theory for a Press disturbance

Our analysis follows the perturbation approach in Chapter 2 of Kato (1976). For a press, We examine the effects of changing the matrix $\mathbf{B}$ by a small amount to $\mathbf{B} + \epsilon\mathbf{D}$. This disturbance in the matrix elements of $\mathbf{B}$ at time t_0 will produce changes in the dominant eigenvalue and corresponding right eigenvector as,

$$\lambda(\epsilon) = \lambda_0 + \epsilon\,\eta_1 + \epsilon^2\,\eta_2 + O(\epsilon^3), \tag{S2}$$

$$u(\epsilon) = \mathbf{u}_0 + \epsilon\,\mathbf{z}_1 + \epsilon^2\,\mathbf{z}_2 + O(\epsilon^3), \tag{S3}$$

so that

$$(\mathbf{B} + \epsilon\mathbf{D})\,(\mathbf{u}_0 + \epsilon\,\mathbf{z}_1 + \epsilon^2\,\mathbf{z}_2) \approx (\lambda_0 + \epsilon\,\eta_1 + \epsilon^2\,\eta_2)\,(\mathbf{u}_0 + \epsilon\,\mathbf{z}_1 + \epsilon^2\,\mathbf{z}_2). \tag{S4}$$

Multiplying through in equation (S4) and collecting terms in $\epsilon^0, \epsilon^1, \epsilon^2$ leads to three equations,

$$\mathbf{B}\mathbf{u}_0 = \lambda_0\,\mathbf{u}_0, \tag{S5}$$

$$\mathbf{B}\mathbf{z}_1 + \mathbf{D}\mathbf{u}_0 = \lambda_0\mathbf{z}_1 + \eta_1\mathbf{u}_0, \tag{S6}$$

$$\mathbf{B}\mathbf{z}_2 + \mathbf{D}\mathbf{z}_1 = \lambda_0\mathbf{z}_2 + \eta_1\mathbf{z}_1 + \eta_2\mathbf{u}_0. \tag{S7}$$

The first of these is obviously true. The others must be used to find the perturbations – the subject of classical perturbation theory. The first-order effects in the middle

equation (S6) yield the first derivatives of λ_0 and have been applied in ecology Caswell (1996) and Caswell (2001) to study sensitivity and elasticity.

This first-order result is

$$\eta_1 = \mathbf{v}_0^{\dagger}\mathbf{D}\,\mathbf{u}_0, \tag{S8}$$

$$= \sum_i \sum_j v_{0i}\mathbf{D}_{ij}u_{0j}. \tag{S9}$$

Following Caswell, take the (i, j) element of $\mathbf{D}$ to be 1 $(d_{ij} = 0)$ and all other elements to be zero, thus yielding the sensitivity

$$v_{0i}\,u_{0j} = \frac{\partial \lambda_0}{\partial b_{ij}} = s_{ij}. \tag{S10}$$

S2.1 Second Order Perturbation: finding η_2

Second-order perturbations yield η_2. Similar to first-order perturbation, multiply equation (S7) by $\mathbf{v}_0^{\dagger}$ on the left and use orthogonality to see that

$$\eta_2 = \mathbf{v}_0^{\dagger}\mathbf{D}\,\mathbf{z}_1. \tag{S11}$$

Note that η_2 is a nonlinear response (coefficient of ϵ^2 in equation (S7)).

The first-order analysis along with equation (S6) yields

$$\mathbf{z}_1 = \frac{1}{\lambda_0}(\mathbf{I} - \mathbf{Q}_1)^{-1}\,(\mathbf{I} - \mathbf{Q}_0)\mathbf{D}\mathbf{u}_0. \tag{S12}$$

Note that as in the main text, $\mathbf{z}^* = (\mathbf{I} - \mathbf{Q}_0)\mathbf{D}\mathbf{u}_0/\lambda_0$ and so,

$$\mathbf{z}_1 = (\mathbf{I} - \mathbf{Q}_1)^{-1}\mathbf{z}^* = (\mathbf{Q}_1 + \mathbf{Q}_1^2 + \mathbf{Q}_1^3 + \cdots)\mathbf{z}^*. \tag{S13}$$

Note that in the main text $\mathbf{Z} = (\mathbf{I} - \mathbf{Q}_1)^{-1} z^* = \mathbf{z}_1$.

Substitute the value of $\mathbf{z}_1$ in equation (S11) to get

$$\eta_2 = \frac{1}{\lambda_0}\mathbf{v}_0^\dagger\mathbf{D}\,(\mathbf{I} - \mathbf{Q}_1)^{-1}\,(\mathbf{I} - \mathbf{Q}_0)\mathbf{D}\mathbf{u}_0. \tag{S14}$$

Now define transient response matrix (TRM)

$$\mathbf{J}_0 = \frac{1}{\lambda_0}(\mathbf{I} - \mathbf{Q}_1)^{-1}\,(\mathbf{I} - \mathbf{Q}_0). \tag{S15}$$

So finally, the second order coefficient is

$$\eta_2 = \mathbf{v}_0^\dagger\mathbf{D}\,\mathbf{J}_0\mathbf{D}\mathbf{u}_0. \tag{S16}$$

S2.2 Second order derivatives

Write the change in the dominant eigenvalue as a Taylor series to order ϵ^2.

$$\lambda(\mathbf{B} + \epsilon\mathbf{D}) = \lambda_0 \tag{S17}$$

$$+ \epsilon \sum_{ij} \mathbf{D}_{ij} \frac{\partial \lambda_0}{\partial b_{ij}} \tag{S18}$$

$$+ \frac{1}{2}\epsilon^2 \sum_{ij} \sum_{mn} \mathbf{D}_{ij} \, \mathbf{D}_{mn} \frac{\partial^2 \lambda_0}{\partial b_{ij} \partial b_{mn}}. \tag{S19}$$

We remove the order epsilon contribution. Now note that η_2 is the second-order contribution of $O(\epsilon^2)$ contribution ϵ^2. Using this expansion again to order ϵ^2,

$$\lambda(\mathbf{B} + \epsilon\mathbf{D}) - \lambda_0 + \lambda(\mathbf{B} - \epsilon\mathbf{D}) - \lambda_0 = \epsilon^2 \sum_{ij} \sum_{mn} \mathbf{D}_{ij} \, \mathbf{D}_{mn} \frac{\partial^2 \lambda_0}{\partial b_{ij} \partial b_{mn}}$$

Now use equation (S2) and equation (S16) to get

$$2\,\eta_2 = \sum_{ij} \sum_{mn} \mathbf{D}_{ij} \, \mathbf{D}_{mn} \frac{\partial^2 \lambda_0}{\partial b_{ij} \partial b_{mn}}. \tag{S20}$$

Perturbing only the (pq) element yields

$$\frac{\partial^2 \lambda_0}{\partial b_{pq} \partial b_{pq}} = 2\,\eta_2(pq, pq). \tag{S21}$$

Now perturb distinct elements p, q and k, l, to find

$$\frac{\partial^2 \lambda_0}{\partial b_{pq} \partial b_{kl}} = v_{0p} \mathbf{J}_{qk} u_{0l} + v_{0k} \mathbf{J}_{lp} u_{0q} \tag{S22}$$

With the sensitivities, $s_{pl} = v_{0p}\, u_{0l}$ we can write this using Kronecker products as

$$\frac{\partial^2 \lambda_0}{\partial b_{pq} \partial b_{kl}} = \left[\mathbf{S} \otimes \mathbf{J}^T + \mathbf{J}^T \otimes \mathbf{S} \right]_{pl,kq} \tag{S23}$$

Getting to the Hessian - the matrix of second derivatives

Here we construct a matrix, Hessian ($\mathbf{H}$), to place all the second derivatives calculated from equation (S22). Suppose the population matrix $\mathbf{B}$ is $S \times S$. Then define indices x, y that take values $1, 2, \ldots, S^2$. We list the elements of $\mathbf{B}$ columnwise which gives a vector $\mathbf{b}$, so $b_{pq} \equiv \mathbf{b}_x$ with

$$x = p + (q - 1)S, \tag{S24}$$

Therefore,

$$\mathbf{H}_{xy} = \frac{\partial^2 \lambda_0}{\partial b_{pq} \partial b_{kl}}, \tag{S25}$$

where $x = p + (q - 1)S$ and $y = k + (l - 1)S$.

S2.3 The alternative route to calculate the second derivatives

The second route, I to III and then III to IV. At I the SSD is $\mathbf{u}_0$ and the stable reproductive value is $\mathbf{v}_0$, so in going from I to III the fitness changes by

$$g\, v_{0p}\, u_{0q}. \tag{S26}$$

Now we are at III, but there the SSD is $(\mathbf{u}_0 + \mathbf{Z}_2)$ with

$$\mathbf{Z}_2 = f \begin{pmatrix} J_{0,1p} u_{0q} \\ J_{0,2p} u_{0q} \\ \vdots \end{pmatrix}. \tag{S27}$$

Also at III, the stable reproductive value is now $(\mathbf{v}_0 + \mathbf{Y}_2)$ with

$$\mathbf{Y}_2^T = g \begin{pmatrix} J_{0,q1} v_{0p} & J_{0,q2} v_{0p} & \ldots \end{pmatrix}. \tag{S28}$$

So in the transition III to IV, the fitness changes by the product of

a) the stable proportion in stage l, which equation (S27) shows is $(\mathbf{u}_{0l} + \mathbf{Z}_{2l})$,

b) the change in the rate, f,

c) the stable reproductive value in stage k, which equation (S28) shows is $(\mathbf{v}_{0k} + \mathbf{Y}_{2k})$.

Thus the total change in the presses from I to III, and III to IV (see Figure (3) in the main manuscript) is

$$
\begin{aligned}
& g\, v_{0p}\, u_{0q} + f\, (\mathbf{v}_{0k} + \mathbf{Y}_{2k})\, (\mathbf{u}_{0l} + \mathbf{Z}_{2l}) \\
\approx & f\, v_{0k}\, u_{0l} + g\, v_{0p}\, u_{0q} + f\, \left(u_{0l}\, \mathbf{Y}_{2k} + v_{0k} \mathbf{Z}_{2l}\right), \qquad \text{(S29)}
\end{aligned}
$$

where the higher order term $g\mathbf{Y}_{2k}\mathbf{Z}_{2l}$ is ignored because it is close to zero. Split this up into two bits,

$$
\begin{aligned}
\text{Linear change} &= f\, v_{0k}\, u_{0l} + g\, v_{0p}\, u_{0q}, \\
&+ \\
\text{Nonlinear change} &= f\, g\, \left(u_{0q}\, v_{0k}\, J_{0,lp} + u_{0l}\, v_{0p}\, J_{0,qk}\right). \qquad \text{(S30)}
\end{aligned}
$$

Split this up into two bits,

$$
\begin{aligned}
\text{Linear change} &= f\, v_{0k}\, u_{0l} + g\, v_{0p}\, u_{0q}, \\
&+ \\
\text{Nonlinear change} &= f\, g\, \left(u_{0q}\, v_{0k}\, J_{0,lp} + u_{0l}\, v_{0p}\, J_{0,qk}\right). \qquad \text{(S31)}
\end{aligned}
$$

The nonlinear change yields the second derivatives of fitness

$$\frac{\partial^2 \lambda_0}{\partial b_{pq} \partial b_{kl}} = s_{kq} J_{0,lp} + s_{pl} J_{0,qk}, \tag{S32}$$

S2.4 Stepwise Calculation of Second derivatives for *Phaseolus lunatus*

The matrix for *Phaseolus lunatus* is

$$\mathbf{B} = \begin{pmatrix} 0.00 & 0.00 & 0.00 & 0.42 & 28.90 & 104.00 \\ 0.16 & 0.00 & 0.00 & 0.00 & 0.00 & 0.00 \\ 0.12 & 0.14 & 0.00 & 0.00 & 0.00 & 0.00 \\ 0.08 & 0.09 & 0.01 & 0.01 & 0.00 & 0.00 \\ 0.00 & 0.00 & 0.00 & 0.05 & 0.16 & 0.00 \\ 0.00 & 0.00 & 0.00 & 0.00 & 0.18 & 0.25 \end{pmatrix}$$

$$\mathbf{u}_0 = \begin{pmatrix} 0.645 \\ 0.134 \\ 0.125 \\ 0.086 \\ 0.007 \\ 0.002 \end{pmatrix} \& \ \mathbf{v}_0 = \begin{pmatrix} 0.373 \\ 0.340 \\ 0.037 \\ 2.842 \\ 39.929 \\ 74.918 \end{pmatrix}$$

$$
\mathbf{J}_0 = \begin{pmatrix}
0.5573 & -0.6256 & -0.0675 & -2.7940 & -10.3566 & 18.6310 \\
0.0509 & 1.1128 & -0.0206 & -1.0802 & -9.1522 & -9.2380 \\
0.0355 & 0.0497 & 1.2824 & -1.0979 & -9.8089 & -11.0066 \\
0.0232 & 0.0283 & 0.0032 & 0.5605 & -6.8290 & -7.7534 \\
-0.0024 & -0.0016 & -0.0002 & 0.0131 & 0.6202 & -1.5080 \\
-0.0026 & -0.0022 & -0.0002 & -0.0089 & 0.0264 & 1.0525
\end{pmatrix}
$$

To obtain the second derivative of growth rate, we need the marginal effect of perturbing two demographic rates. For ease of exposition, say we make a press disturbance and change (2% of the element's value) the $(1, 5)$ and $(6, 5)$ matrix elements to $b_{15} + 0.578$ and $b_{65} + 0.0036$, where the change $g = 0.578$ and $f = 0.0036$.

Figure (3) in the main manuscript illustrates two distinct ways of carrying out the above press disturbance, which must lead to the same overall change in fitness.

Say we use the first route, A to B and then B to D. In the change from A to B, our simple argument above shows that the change in fitness is the product

$$
f\, v_{06}\, u_{05} = 0.0036 \times 74.9 \times 0.007 = 1.89 \times 10^{-3}. \tag{S33}
$$

Now we want to make the change from B to D. But at B, the SSD has already changed to $(\mathbf{u}_0 + f\,\mathbf{Z}_1)$ with

$$\mathbf{Z}_1 = u_{05} \begin{pmatrix} J_{0,16} \\ J_{0,26} \\ J_{0,36} \\ J_{0,46} \\ J_{0,56} \\ J_{0,66} \end{pmatrix} = 0.007 \times \begin{pmatrix} 18.6 \\ -9.2 \\ -11.0 \\ -7.8 \\ -1.5 \\ 1.1 \end{pmatrix} = \begin{pmatrix} 0.13 \\ -0.06 \\ -0.08 \\ -0.05 \\ -0.01 \\ 0.01 \end{pmatrix}. \tag{S34}$$

Also at B, the stable reproductive value has also already changed to $(\mathbf{v}_0 + f\,\mathbf{Y}_1)$ with

$$\begin{aligned} \mathbf{Y}_1^T &= v_{06} \begin{pmatrix} J_{0,51} & J_{0,52} & J_{0,53} & J_{0,54} & J_{0,55} & J_{0,56} \end{pmatrix} \\ &= 74.92 \times \begin{pmatrix} -0.0024 & -0.0016 & -0.0002 & 0.0131 & 0.6202 & -1.5080 \end{pmatrix} \\ &= \begin{pmatrix} -0.2 & -0.1 & 0.0 & 1.0 & 46.5 & -113.0 \end{pmatrix}. \end{aligned} \tag{S35}$$

Now we are ready to use our approach to say that in the transition B to D, the fitness changes by the product of

a) the stable proportion in stage 5, which equation (S34) shows is $(u_{05} + fZ_{15}) = 7.02 \times 10^{-3}$,

b) the change in the rate, $g = 0.578$,

c) the stable reproductive value in stage 1, which equation (S35) shows is $(v_{01} + fY_{11}) = 0.372$.

The product of these terms has to be added to the change (equation (S33)) to get the total change in growth rate A to B to D,

$$f\, v_{06}\, u_{05} + d\, v_{01}\, u_{05} + f\, d\, \left(u_{05}\, Y_{11} + v_{01} Z_{15}\right) = 3.41 \times 10^{-3}. \qquad \text{(S36)}$$

Using (S34 – S35) the total change is the sum

$$\text{Linear change} = f\, v_{06}\, u_{05} + g\, v_{01}\, u_{05} = 3.42 \times 10^{-3},$$

$$+$$

$$\text{Nonlinear change} = f\, g\, \left(u_{05}\, v_{06}\, J_{0,15} + u_{05}\, v_{01}\, J_{0,56}\right) = -1.09 \times 10^{-5}. \qquad \text{(S37)}$$

As shown in Figure (3) in the main manuscript, we could alternatively go from A to C and then C to D. That process involves distinct changes to the SSD and reproductive value. But we get the same final result as in equation (S37).

We conclude that the nonlinearity is revealed by making two press disturbances. Think about these changes in terms of the second derivatives of fitness to find

$$\frac{\partial^2 \lambda_0}{\partial b_{15} \partial b_{65}} = \left[s_{15}\, J_{0,56} + s_{65}\, J_{0,51}\right] = -0.005$$

where we have used the sensitivities equation (12) (in the main manuscript). Note that our expression for the second derivative is symmetric with respect to an exchange of the elements b_{15}, b_{65} (as it should be).

The curvature of fitness as measured by the second derivatives depends on TRM ($\mathbf{J}_0$). Consequently any analysis of second derivatives will provide detailed information about TRM. The next section describes the many connections between TRM and transient dynamics.

Section S3: When the population matrix has distinct eigenvalues

Write $\mathbf{i} = \sqrt{-1}$, and the higher eigenvalues as

$$\lambda_j = e^{(r_j + \mathbf{i}\omega_j)},\ j \geq 1,$$
$$\frac{\lambda_j}{\lambda_0} = \rho_j\, e^{\mathbf{i}\omega_j},\ j \geq 1,\ \text{with } \rho_j < 1.$$

The ratios $\rho_j = \lambda_j/\lambda_0 < 1$ are called the damping ratios. Each eigenvalue has a corresponding right, left eigenvector, $\mathbf{B}\mathbf{u}_j = \lambda_j \mathbf{u}_j$, $\mathbf{v}_j^{\dagger}\mathbf{B} = \lambda_j \mathbf{v}_j^{\dagger}$, and we set $(\mathbf{v}_j, \mathbf{u}_j) = \mathbf{v}_j^{\dagger}\mathbf{u}_j = 1$ (the superscript † indicates the combination of a transpose and a complex conjugate). Then we can write a spectral decomposition (Good 1969) of the matrix $\mathbf{B}$ as

$$\mathbf{B} = \lambda_0\, \mathbf{u}_0\, \mathbf{v}_0^{\dagger} + \lambda_1\, \mathbf{u}_1\, \mathbf{v}_1^{\dagger} + \lambda_2\, \mathbf{u}_2\, \mathbf{v}_2^{\dagger} + \ldots.$$

Recalling the definition of $\mathbf{Q}_1 = \mathbf{B} - \frac{\mathbf{Q}_0}{\lambda_0}$, we find

$$\mathbf{Q}_1 = \sum_{j \geq 1} \rho_j \, e^{\mathbf{i}\omega_j} \, \mathbf{u}_j \, \mathbf{v}_j^\dagger.$$

Use a geometric series expansion to find the explicit expression

$$\mathbf{J}_0 = \sum_{j \geq 1} \frac{\mathbf{u}_j \, \mathbf{v}_j^\dagger}{\left(1 - \rho_j \, e^{\mathbf{i}\omega_j}\right)}.$$

This happens to be a spectral decomposition of $\mathbf{J}_0$, excluding the eigenvector $\mathbf{u}_0$ for which the eigenvalue is 0. Thus for $j = 1, 2, \ldots$ the vectors $\mathbf{u}_j \, \mathbf{v}_j$ are right, left eigenvectors of $\mathbf{J}_0$ corresponding to eigenvalue $\left(1 - \rho_j \, e^{\mathbf{i}\omega_j}\right)^{-1}$.

Section S4: Relation between TRM and Cohen's cumulative distance

$$
\begin{aligned}
\mathbf{J}_0 - \mathbf{D}_1 &= (\mathbf{I} - \mathbf{Q}_1)^{-1} (\mathbf{I} - \mathbf{Q}_0) - (\mathbf{I} - \mathbf{Q}_1)^{-1} + \mathbf{Q}_0 \\
&= (\mathbf{I} - \mathbf{Q}_1)^{-1} (\mathbf{I} - \mathbf{Q}_0 - \mathbf{I}) + \mathbf{Q}_0 \\
&= \mathbf{Q}_0 - \mathbf{Q}_0 (\mathbf{I} - \mathbf{Q}_1)^{-1} \\
&= \mathbf{Q}_0 (\mathbf{I} - \mathbf{Q}_1) (\mathbf{I} - \mathbf{Q}_1)^{-1} - \mathbf{Q}_0 [\mathbf{I} - \mathbf{Q}_1)^{-1} \\
&= ((\mathbf{I} - \mathbf{Q}_1) \mathbf{Q}_0 - \mathbf{Q}_0) (\mathbf{I} - \mathbf{Q}_1)^{-1} \\
&= (\mathbf{Q}_0 - \mathbf{Q}_1\mathbf{Q}_0 - \mathbf{Q}_0) (\mathbf{I} - \mathbf{Q}_1)^{-1} \\
&= 0 \text{ (because } \mathbf{Q}_1\mathbf{Q}_0 \text{ are orthogonal as discussed above)}
\end{aligned}
$$

References

Caswell, H. (2001). *Matrix population models: construction, analysis and interpretation*. 2nd. Sunderland, Mass.: Sinauer associates, Sunderland, Mass.

Caswell, Hal (1996). “Second derivatives of population growth rate: calculation and applications”. *Ecology*, pp. 870–879.

Good, Irving John (1969). “Some applications of the singular decomposition of a matrix”. *Technometrics* 11.4, pp. 823–831.

Kato, Tosio (1976). *Perturbation theory for linear operators*. Vol. 132. Springer Science & Business Media.
